# Magnetic Triggering of Functional Organosilica Filler Particles for Controlling the Thermoreversible Attachment to Polymer Matrices

**DOI:** 10.1021/acs.langmuir.4c02589

**Published:** 2024-10-30

**Authors:** Valentin Hagemann, Laura Finck, Felix Klempt, Patrick Evers, Florian Nürnberger, Irene Morales, Nina Ehlert, Philipp Junker, Peter Behrens, Henning Menzel, Sebastian Polarz

**Affiliations:** † Institute of Inorganic Chemistry, Leibniz-University Hannover, Callinstrasse 9, 30167 Hannover, Germany; ‡ Lower Saxony Center for Biomedical Engineering, Implant Research and Development (NIFE), Stadtfelddamm 34, 30625 Hannover, Germany; § Institute for Technical Chemistry, Technical University Braunschweig, Hagenring 30, 38106 Braunschweig, Germany; ∥ Institute of Continuum Mechanics, Leibniz-University Hannover, An der Universität 1, 30823 Garbsen, Germany; ⊥ Institute of Materials Science, Leibniz-University Hannover, An der Universität 2, 30823 Garbsen, Germany

## Abstract

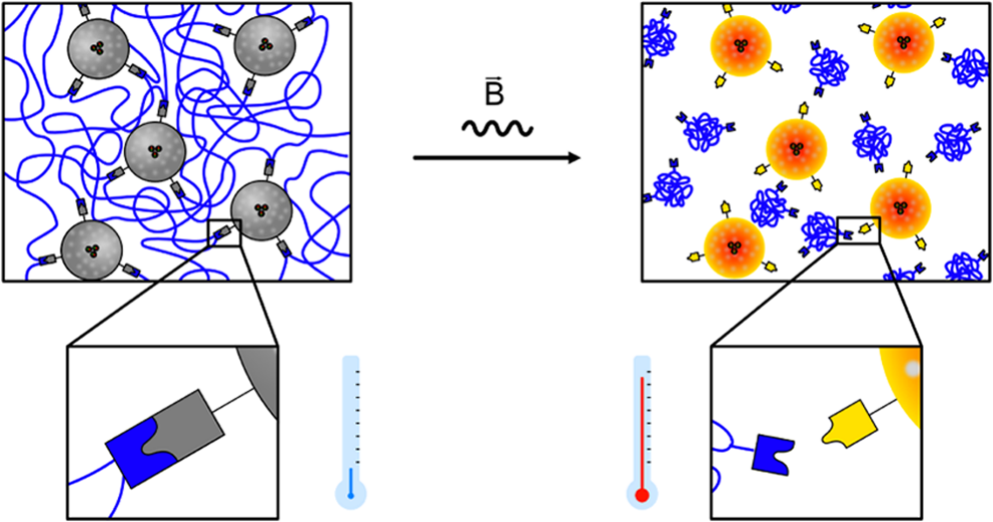

Polymer materials containing filler particles are widely used in automotive components, construction materials, packaging materials, medical devices and supplies, and much more. The fillers strongly modulate the properties of the composite. In some applications, one is interested in smart features of those composites, meaning one can post-synthetically and reversibly change the characteristics as a response to an easy-to-apply trigger. For example, if the excision of an implant material may become necessary, then the polymer–filler hybrid changing from mechanically robust to soft(er) would be very beneficial. Here, we present a proof-of-concept study that shows that stimuli-responsive polymer–filler composites can be achieved by functional organosilica nanoparticles. The nanoparticles comprise a superparamagnetic core surrounded by a mesoporous organosilica shell. The polymer matrix is attached to the filler via Diels–Alder coupling to maleimide groups present at the surface of the organosilica. Exposure to an alternating magnetic field generates local heat in the organosilica particles. Utilizing fluorescence probes bound to the polymer backbone’s side chains, we could prove that detachment occurs via a retro-Diels–Alder reaction within minutes.

## Introduction

Polymers exhibit diverse mechanical properties that make them suitable for numerous applications, depending on their structure and composition. These properties are influenced by the polymer’s molecular weight, degree of crystallinity, and the nature of the monomer units. However, one of the most important methods for tuning the mechanical properties of polymers is by incorporating fillers. They are added to improve the mechanical strength, stiffness, and thermal stability and reduce costs by replacing more expensive base polymers. The nature of the filler and how the particles are linked to the polymer matrix is crucial for the change in properties compared to the pure plastic material.^1^ Among the common fillers like carbon black or calcium carbonate, silica is in a prime position and is produced at the multiton scale for this purpose.^2^ Jawaid et al. have discussed the potential of nanoscaled particles as fillers^3^ and pointed out the importance of silica nanoparticles for this particular application.^4^

The charm of silica nanoparticles is that one can engineer them in size, porosity, chemical composition, and associated surface functionalities.^5^ Mesoporous silica particles have been used much more seldomly as fillers than compact particles despite the advantageous properties in the composites, as pointed out in several articles.^6,7^ The latter is surprising because there is a large knowledge base on the different possibilities for equipping mesoporous silica particles with organic functionalities.^8^ Organosilane sol–gel precursors R-Si(OEt)_3_ can be used for the modification of surface silanol groups (post-functionalization method), or they can be added directly to the sol–gel process (co-condensation method). Bis-silylated precursors containing a bridging organic group (EtO)_3_Si–R–Si(OEt)_3_ are special as they lead to the so-called periodically ordered mesoporous organosilicas (PMOs).^9−11^ Because the precursors can be used in a pure form, PMOs possess a high content of the organic functionalities RSi_2_O_3_, making them attractive for various applications.^11^ Besides their use as colloids in the biomedical and medical field, PMOs were already integrated as fillers into several polymers, like PMMA,^12,13^ PES,^14^ and EVOH^15^ to form functional composites.

**Scheme 1:**
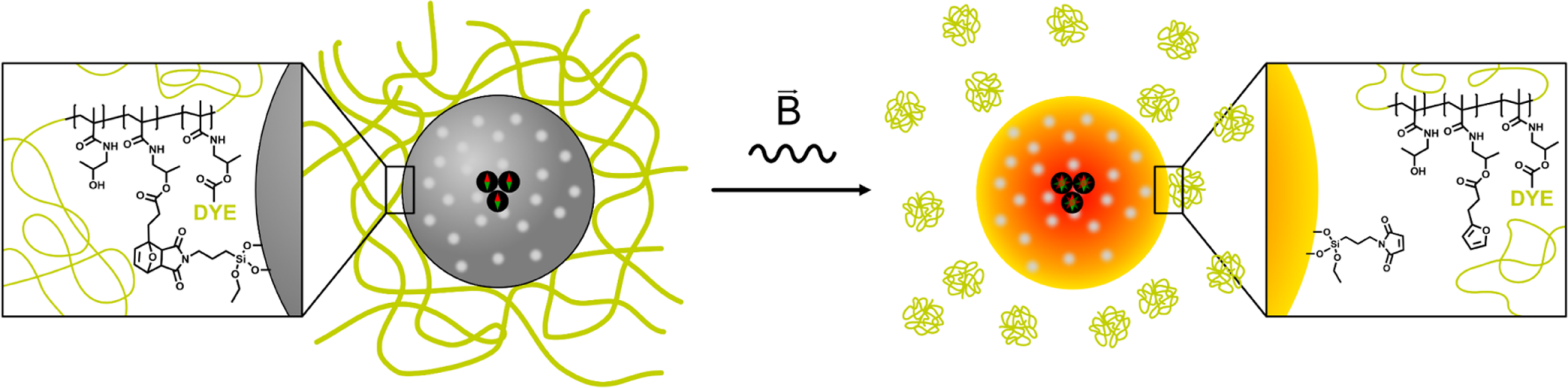
Schematic Illustration of the Thermo-Induced (Gray → Orange) Detachment of Polymers from a PMO Nanoparticle by Retro-Diels–Alder Reaction The particles are equipped with a superparamagnetic core, and heat is generated by applying an alternating magnetic field (B).

Following the aim of creating responsive composite materials, one possibility is to introduce this property to the polymer.^13^ In this case, the adaptation has to be redeveloped for each polymer. A more universal approach is the use of functional filler particles that may enrich a polymer material with the particular feature of reversibly changing its properties after synthesis. Therefore, the concept of the current article is to implement an element of responsiveness into PMO filler particles. Our idea is indicated in Scheme 1. The surface of organosilica nanoparticles is decorated with a maleimide functionality, which can form a Diels–Alder (DA) adduct with a furan group on a side chain of a polymer. The furan/maleimide adduct is known to undergo the retro-DA at moderate temperatures below 100 °C.^16−18^ Heat produced in the filler particles’ internal regions should provoke the polymer’s detachment. Pores connecting to the surface aid in transporting heat to the polymer.

One of the best methods for producing local heat, which is also applied in this approach, is via superparamagnetic iron oxide nanoparticles (SPIONs) placed in an oscillating magnetic field (magnetic hyperthermia).^19,20^ Since SPIONs are catalytically active, exhibit a reactive surface, and tend to aggregate, they are often coated for stabilization.^21−23^ PMO is also available as shell material, combining organic and inorganic material properties.^24^ An organosilica shell on top of the SPION cores enhances stability, biocompatibility, and functionalization potential. The organosilica shell acts as a barrier against aggregation and oxidation, thus improving the colloidal stability and shelf life of the SPIONs.

This work presents a novel approach for creating a polymer–nanoparticle composite that integrates functional organosilica nanoparticles with superparamagnetic cores, allowing a change in the material properties using a magnetic trigger. By the bond cleavage of the Diels–Alder reaction between maleimide-modified organosilica and furan-functionalized polymers, the mechanical properties of the composite can be controlled through magnetic hyperthermia. By destroying the bonding between filler and polymer, a softening of the material should occur, which can be useful if the excision of an implant may become necessary. This method marks a significant improvement over traditional composites by enabling post-synthetic adjustments, making it particularly interesting for biomedical applications where non-invasive modifications are advantageous and very local heating is necessary to avoid tissue damage. Here, we present the first step of developing this material, concentrating on the attachment and detachment of the polymer.

The paper is organized as follows: First, we describe the magnetite cores used and coat them with a PMO shell. Next, the polymer is attached. We then focus on magnetic hyperthermia and question whether the temperature at the surface of the filler particles becomes high enough to initiate the retro-DA.

## Materials and Methods

### Chemicals

All chemicals were used without further purification. Iron(II) chloride tetrahydrate (≥99%), iron(III) chloride hexahydrate (99%), sodium citrate tribasic dihydrate (≥99%), cetyltrimethylammonium bromide (CTAB, ≥98%), ammonium hydroxide solution (≥25% NH_3_ in water), 1,4-bis(triethoxysilyl)-benzol (BTEB, 96%), toluene (anhydrous, 99.8%), absolute ethanol (≥99.5%), ammonium nitrate (≥99%), 2,2′-azobis(2-methylpropionitril) (AIBN, ≥98%), *N*,*N*′-dicyclohexylcarbodiimide (DCC, ≥99%), and dimethyl sulfoxide (DMSO, ≥99%) were purchased from Sigma-Aldrich Corporation (München, Germany). 1-[3-(Triethoxysilyl)propyl]-1*H*-pyrrole-2,5-dione (maleimide silane, 95%) was purchased from Astatech (Bristol, USA). Dichloromethane (DCM, ≥99%) and diethyl ether (ET_2_O, ≥99%) were purchased from Fisher Scientific (Hampton, United States). Dimethyaminopyrridin (DMAP, ≥98%) was purchased from TCI (Eschborn, Germany). 3-(2-Furyl) propionsäure (97%) was purchased from Alfa Aesar (Haverhill, United States). *N*-(2-Hydroxypropyl)methacrylamide (HPMA) was purchased from Polyscience (Warrington, United States).

### Synthesis of Magnetic PMOs (mPMOs) in Different Particle Sizes

In the first step, the citrate-stabilized magnetite nanoparticles (SPIONs) were synthesized according to the literature.^22^ The SPIONs (15, 30, or 60 mg) were dispersed for 15 min in 90 μL of water and 7.2 mL of ethanol, using an ultrasonic bath. Then, 28 mL of water, 1.4 mL of ammonium hydroxide solution, and 4.2 mL of 0.11 M CTAB solution in water/ethanol (2:1) were added. The reaction mixture was shaken vigorously for 2 h at 300 rpm. Then, 157 μL of BTEB were added and the mixture was shaken for 24 h at room temperature at 300 rpm and afterward placed in the convection oven at 80 °C for another 24 h. The PMOs were collected by centrifugation, washed three times with ethanol, and dried under vacuum. The surfactant CTAB was removed by extraction in 30 mL of ethanol and 0.17 g of ammonium nitrate for 2 h at 80 °C. Again, the extracted particles were collected by centrifugation at 16 000*g*, washed three times with ethanol, and dried under vacuum.

### Modification of Magnetic PMOs with Maleimide Silane

The particles were modified with the maleimide silane by a post-grafting method. To do so, 100 mg of the magnetic PMOs were dispersed in 30 mL of anhydrous toluene for 10 min using an ultrasonic bath and stirred under nitrogen atmosphere. Then, 40 mg of the maleimide silane was added, and the reaction mixture was heated to reflux and stirred overnight. The particles were collected by centrifugation at 16 000*g*, washed three times with ethanol, and dried under vacuum.

### Synthesis of P-HPMA-Furan-Fluorescein

The synthesis of poly(*N*-(2-hydroxy-propyl)methacrylamide) (P-HPMA) and subsequent functionalization with furan was performed as described before with an adjustment of the polymerization parameters to 65 °C and 0.5 mol % azobis(isobutyronitrile) (AIBN).^25^ The resulting polymer had a functionalization of 4%. For further functionalization of the polymer with fluorescein (0.75 equiv according to OH groups of P-HPMA), the same procedure was used as for functionalization with furan.

### Binding of P-HPMA-Furan-Fluorescein to PMOs by Diels–Alder

The polymer was attached to the magnetic PMOs by a Diels–Alder reaction. For that, 50 mg of maleimide-modified magnetic PMOs were dispersed in 15 mL of water for 10 min, using an ultrasonic bath. In parallel, 10 mg of the polymer were dissolved in 15 mL of water. Since some of the maleimide groups are located within the pores and thus not accessible to the polymer, a 10:1 excess of maleimide groups relative to the furan groups on the polymer was selected. This should ensure that there are sufficient opportunities for binding between the polymer and the particles. The polymer solution was then added to the nanoparticle dispersion and stirred for 72 h at 40 °C. The particles were again collected by centrifugation at 16 000*g*, washed two times with water and two times with ethanol, and dried under vacuum.

### Detachment of P-HPMA-Furan-Fluorescein from PMOs by Retro-Diels–Alder

The detachment of H-HPMA polymer by applying high-frequency magnetic fields has been done with a magneTherm RC system from nanoTherics Ltd. (Warrington, United Kingdom) which allows to work with 10 different resonance frequencies from 100 to 900 kHz and a maximum applied field of 30 mT, with a coil of 50 mm diameter and 18 turns. By combination of the coil with the appropriate capacitor (in which the nanoparticle colloid showcased the highest heating efficiency), a frequency of 624 kHz and an applied field of 12 mT were selected and applied to the sample for 60 min. The coil temperature was maintained at 20 °C with a water-circulating bath, and the sample was introduced in an insulated polystyrene holder to avoid any heat loss. Finally, the temperature increase of the magnetic colloid as a function of time has been measured by placing a fiber optic temperature probe in the center of the sample and recorded by means of the magneTherm Software, which also allowed the automated resonance tuning. After the AMF exposure time of 60 min, the particles were redispersed using ultrasonication and afterward magnetically separated, using a magnetic separator DynaMag15 from Invitrogen (Carlsbad, United States) so that 700 μL of the supernatant could be pipetted off. For comparison, a dispersion of 5 mg of polymer-modified nanoparticles dispersed in 1 mL of water was kept for 60 min at room temperature, and another particle dispersion was placed in an oven at 100 °C for 60 min. Again, the particles were redispersed using ultrasonication and afterward magnetically separated and 700 μL of the supernatant were pipetted off and from each sample, three times 200 μL were measured. Afterward, the rest of the supernatant was pipetted off without further processing and the remaining particles were dried under vacuum.

### Measurement and Characterization

Transmission electron microscopy (TEM) was used to investigate the size and morphology of the particles. The measurements were performed on a Hitachi HT7800 at 100 kV (Hitachi Ltd. Cooperation, Chiyoda, Japan). The nanoparticles were dispersed in ethanol by using ultrasonication. A dispersion droplet of 10 μL was put on a holey carbon film copper grid (MicrotoNano, Harlem, the Netherlands) and dried at room temperature. Images were recorded with a CMOS camera (14 bit 5120 × 3840 pixels emsis Xarosa) and acquired by RADIUS 2 imaging software. The particle sizes were determined using an NIH ImageJ.

To prove successful modification of the nanoparticles, FTIR spectroscopy was carried out with the attenuated total reflectance (ATR) unit UATR Accessory from PerkinElmer Spectrum (PerkinElmer, Inc., Waltham, United States) with a DTGS detector. The IR spectra were recorded in the range of 4000–400 cm^–1^ with a resolution of 5 cm^–1^, baseline corrected, and normalized using the PerkinElmer Spectrum 10 software.

Nitrogen physisorption was measured on a 3Flex instrument from Micromeritics (Micromeritics Instrument Corporation, Norcross, United States). Therefore, the samples were outgassed under vacuum for 24 h at 100 °C. Surface areas, pore sizes, and the total pore volume were calculated with MicroActive (version 6.0) software from Micromeritics. For the calculation, the Brunauer–Emmet–Teller (BET) equation and density functional theory (DFT) were applied. The experimental data were fitted to MicroActive Kernel “DFT-Cylinder-N2-Cylindrical Pores-Oxide Surface”.

A Zetasizer type Nano ZS from Malvern Instruments (Malvern, UK) was used for dynamic light scattering and zeta potential measurements, and therefore, the sample was dispersed in water.

^1^H-NMR spectra were performed on a Bruker Biospin Avance III 400 (Rheinstetten, Germany).

UV/vis spectroscopy of furan- and fluorescein-modified P-HPMA polymer were measured on a Spark 10 M (Tecan Trading AG, Männedorf, Switzerland). To determine the furan and fluorescein content, a calibration with isopropyl-3-(2-furyl)propanoate or fluorescein was employed.

Fluorescence intensity measurements were also performed on the Spark 10M, using an excitation wavelength of 485 nm and a bandwidth of 20 nm, and the emission was recorded at 520 nm with a bandwidth of 10 nm.

The magnetic measurements were performed in a SQUID MPMS3 (superconducting quantum interference device) from Quantum Design (Pfungstadt, Germany). The samples were measured in powder in polypropylene holders (QDC-4096-388) and mounted into a brass half-tube sample holder. The hysteresis cycles were performed in VSM mode at 300 K by applying a DC magnetic field of ±5 T.

## Results and Discussion

### Magnetite Core

The fabrication of core–shell nanoparticles started with the synthesis of magnetite nanoparticles via the co-precipitation method described in the literature.^22^ The quality of the obtained particles was demonstrated by dynamic light scattering (DLS) measurements, transmission electron microscopy (TEM), Fourier transform infrared spectroscopy (FT-IR), and superconducting quantum interference device (SQUID) measurement; data shown in Supporting Information Figure S1. Polydisperse nanoparticles with an average size of 20 nm were obtained. One expects superparamagnetic behavior, which is confirmed by SQUID. A magnetic saturation of 70 emu g^–1^ is reached. The IR spectrum is also in agreement with the bands expected for SPIONs.^22^

### Organosilica Shell SPION-Core Nanoparticles

**Figure 1:**
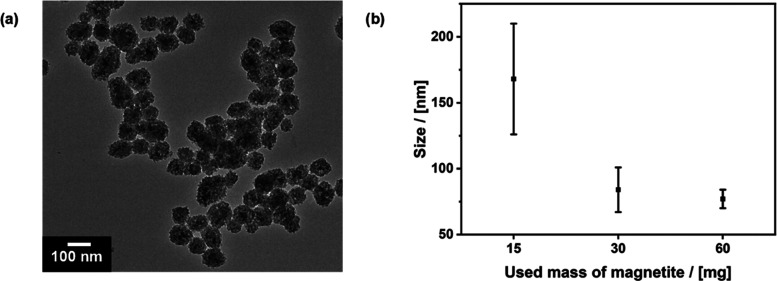
(a) TEM image of core–shell nanoparticles synthesized using 60 mg of magnetite particles as seeds. (b) Correlation between the mass of magnetite used and the thickness of the organosilica shell.

The prepared SPIONs are used as seeds during the sol–gel process, with 1,4-bis(triethoxysilyl)-benzol as a sol–gel precursor (see the experimental part). Figure 1a shows a TEM micrograph of the resulting sample and demonstrates the successful coating of SPIONs with the PMO shell. The organosilica shell is highly porous, confirmed by nitrogen physisorption isotherms as an independent method (see Supporting Information Figures S2 and S4). The samples possess a high specific surface area of 800 cm^2^ g^–1^ and a broad pore-size distribution ranging from 2 to 10 nm.

A decrease in the number of seeds likely leads to a decrease in the thickness of the PMO shell. This is exactly what we see in TEM and DLS data (Supporting Information Figures S2 and S3). Using different amounts of the SPIONs per synthesis batch (*A*: 15 mg, *B*: 30 mg, *C*: 60 mg), the size of the produced core–shell nanoparticles drops from A (168 ± 42 nm) to B (84 ± 17 nm) and C (77 ± 7 nm); Figure 1b. As the particles with the thinnest shell have the highest heat transfer, as later shown in Figure 4b, the PMO C particles are used for most of the following experiments.

The next step is the attachment of maleimide functionalities to the external surfaces of the PMO-SPIONs via post-functionalization with 1-[3-(triethoxysilyl)propyl]-1H-pyrrole-2,5-dione (≅maleimide silane). The successful modification was proven by various methods including FT-IR and thermogravimetric analysis (TGA) and is summarized in Supporting Information Figure S5.

### Polymer Constituent and Attachment

**Figure 2:**
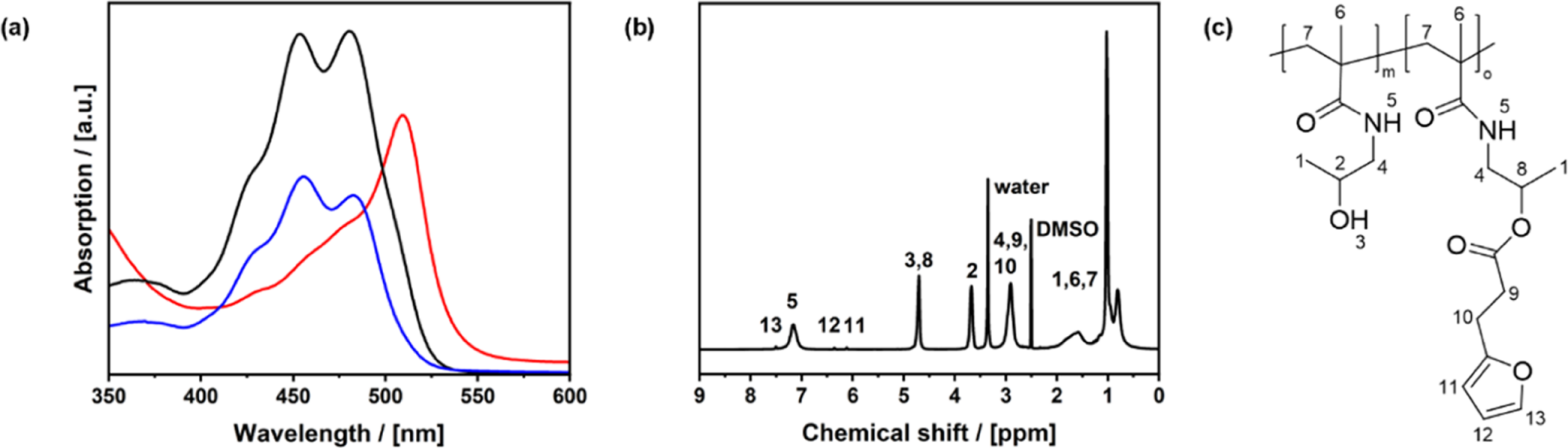
(a) UV/vis spectra of a fluorescein-modified 1 mg mL^–1^ P-HPMA solution in ethanol (red), showing an absorption maximum of 496 nm. The spectra of pure fluorescein (black) and a mixture of fluorescein and P-HPMA (blue) show absorption maxima at 481 and 453 nm. (b) ^1^H-NMR of the side-chain-modified P-HPMA with an assignment to the chemical shifts of the molecule (c).

Poly(N-(2-hydroxy-propyl)methacrylamide) (P-HPMA) was modified with furan. The polymer was now ready for attachment to the PMO-SPIONs. However, we asked ourselves for an effective way to prove the detachment of the polymer when AMF is applied (Scheme 1). In addition to the furan group, a fluorescent dye (fluorescein) was also attached by using an ester link. The successful modification with fluorescein was proved by UV/vis measurements (Figure 2a), showing an absorption maximum at λ_max_ = 496 nm for the fluorescein-modified P-HPMA. In contrast, the spectrum of a mixture of P-HPMA and fluorescein exhibits two maxima at 481 and 453 nm.^26^ This shift indicates that fluorescein successfully bound fluorescein. The fluorescein content was determined to be less than 1% using UV/vis spectroscopy. NMR spectra confirmed the modification with furan groups, as can be seen by the assignment to the chemical shifts of the molecule (Figure 2b,c).

**Figure 3:**
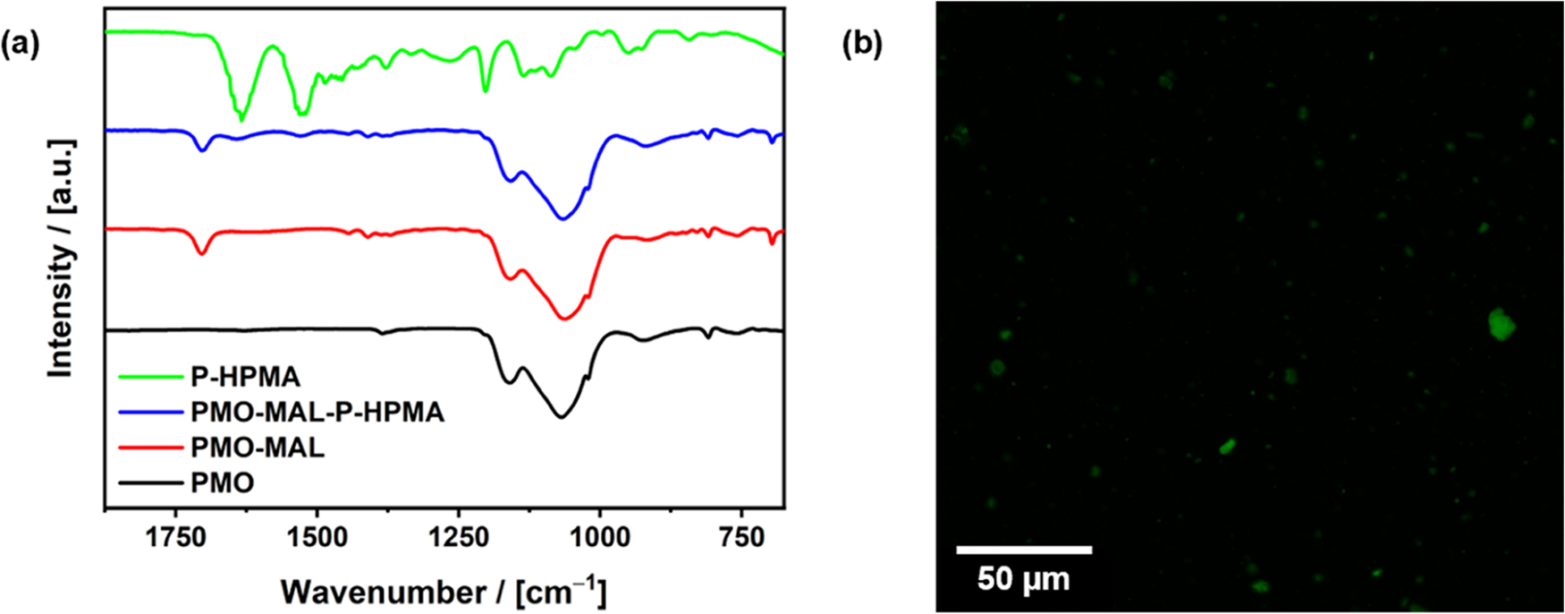
(a) FTIR spectra of magnetic PMOs (PMOs, black), after modification with maleimide (PMO-MAL, red), and after attachment of the P-PHMA-furan-fluorescein polymer (PMO-MAL-P-PHMA, blue). Additional vibration bands occur after every reaction step. (b) Fluorescence microscopy image of the PMO-MAL-P-HPMA particles.

Next, the described polymer was added to a dispersion of PMO-SPION (for details, see the experimental part). The product was separated from the solution by centrifugation and analyzed. Figure 3a compares different FTIR spectra to each other: PMO-SPIONS before and after the modification with the maleimide silane and after the attachment of the P-HPMA-furan-fluorescein polymer (PMO-MAL-P-HPMA, blue). In each spectrum, the dominant vibration band is the Si–O–Si absorption around 1070 cm^–1^, typical for PMO materials. The Fe–O stretching vibration at around 550 cm^–1^ and the Si–C stretching vibration at 520 cm^–1^ are visible. After the consecutive modification with the maleimide silane, the carbonyl stretching band vibration at 1704 cm^–1^ and the maleimide ring deformation band at 694 cm^–1^ occur and prove successful modification. Furthermore, as they are the dominant bands in the P-HPMA FTIR spectra, the arising amide I and II bands at 1640 and 1530 cm^–1^ confirm the following attachment of the P-HPMA-furan-fluorescein. The additional organic content of the samples is detected by TGA as well (Supporting Information Figure S5). Compared to the unmodified particles with a mass loss of 25%, the maleimide-modified nanoparticles exhibit a mass loss of 30% evoked by the additional organic groups on the surface. After polymer attachment, the mass loss is further increased to 34%. Considering the average molecular weight of the polymer (*M*_W_ = 52 730 g mol^–1^; *M*_N_ = 26 470 g mol^–1^), one can conclude that there are only a few polymer chains bound to the surface of the particle probably because each attached macromolecule is not in a stretched conformation and it sterically blocks many maleimide groups. In agreement with the successful modification also, the pore volume and the specific surface area of the material drop; see also Supporting Information Figure S5. Finally, also fluorescence microscopy images (λ_ex_ = 488 nm) show the characteristic emission of the attached fluorescein constituent (λ_em_ = 520 nm; Figure 3b).

### Magnetothermic Properties and Polymer Detachment

**Figure 4:**
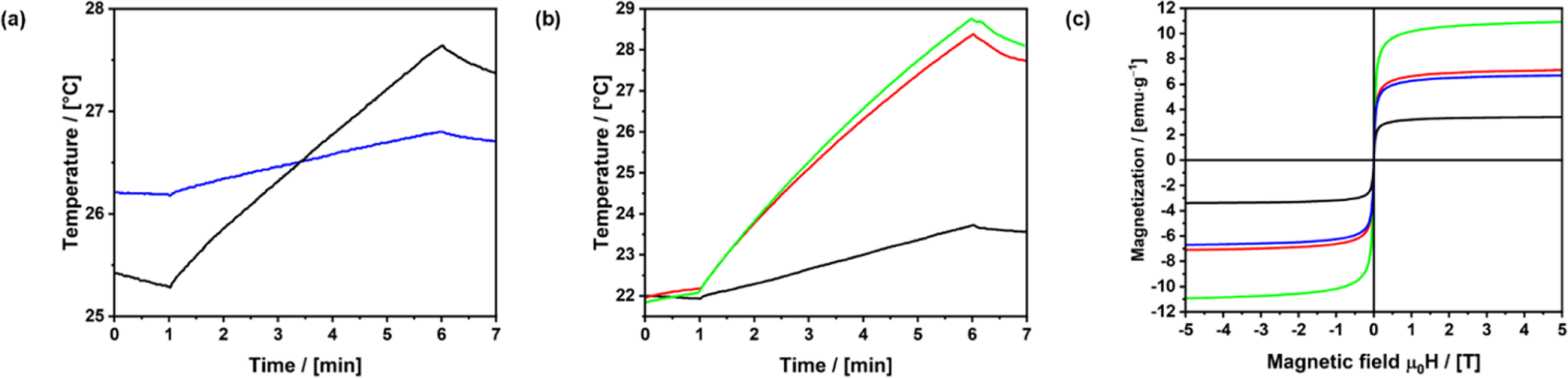
(a) Heating curves of 5 mg of magnetic PMO particles dispersed in 1 mL of water at 30 mT and 107 kHz (blue line) and at 12 mT and 624 kHz (black line). (b) Heating curves of 5 mg PMO A (black line), B (red line), and C (green line) at 12 mT and 624 kHz. (c) SQUID measurements of PMO A (black line), B (red line), C (green line), and after the attachment of P-HPMA to PMO C (blue line). The magnetization is calculated by considering the total sample mass.

Different parameters were explored to determine the frequency and strength of the alternating magnetic field at which the heating of the particles is highest. To determine the efficacy of heating, the macroscopic temperature of the system was used. Initially, a field strength of 30 mT at 107 kHz was applied. Here, only a heating up of less than 1 °C within the first 5 min after applying the field was measured. At a frequency of 624 kHz and a field strength of 12 mT, a change of 2.3 °C was already measured after 5 min, which is why this frequency was used for the polymer detachment in the following experiments. The heating curves are shown in Figure 4a. In Figure 4b, the heating curves of the differently-sized particles are presented. PMO A (Figure 4b, black line), which has the thickest shell (168 ± 42 nm), shows the lowest temperature increase after 5 min of AMF exposure. The temperature increase of PMO C (Figure 4b, green line) is significantly higher since the shell is the thinnest (77 ± 7 nm). PMO B (Figure 4b, red line) has almost a similar shell thickness (84 ± 17 nm) as PMO C leading to a comparable temperature increase. As the particle shell thickness decreases, the magnetic content increases. It is important to note that while the bulk temperature of the solution remains low, simulations and literature confirm that the temperature at the surface of the magnetic particles can be significantly higher, which is sufficient to trigger the retro-Diels–Alder reaction and induce polymer detachment.

The increase in the magnetic content when the shell thickness decreases is also evident from the SQUID measurements when comparing the maximum magnetization at 5 T. For PMO A (Figure 4c, black line), which has the thickest shell, the maximum magnetization at 5 T is the lowest. Conversely, for PMO C (Figure 4c, green line), which has the thinnest shell, the highest magnetization is observed. Furthermore, after the attachment of P-HPMA to PMO C (Figure 4c, blue line), the magnetic content also decreases, leading to a reduction in the maximum magnetization.

**Figure 5:**
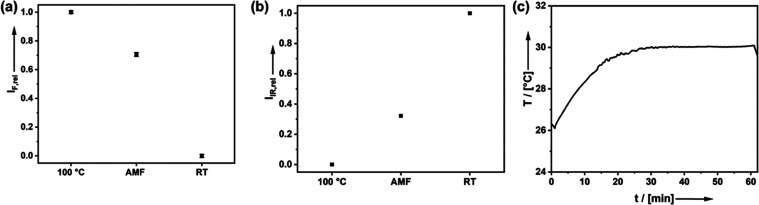
(a) Relative intensity of the fluorescence signal indicating free polymer present in solution after particle separation for three samples: PMO-MAL-P-HPMA treated at 100 °C for 1h (reference sample), kept at RT (reference sample), and exposed to AMF for 1 h. (b) Relative intensity of the IR amide I band at 1641 cm^–1^ measured for the separated PMO-SPIONs for the same set of samples. (c) Heating curve of 5 mg of PMO-MAL-P-HPMA dispersed in 1 mL of water was measured under 1 h AMF exposure at 624 kHz and 12 mT.

After the optimal settings regarding the frequency and field strength of the AMF were determined, the polymer release was investigated. For this purpose, 5 mg of polymer-modified nanoparticles were dispersed in 1 mL of water and exposed to the AMF for 1 h. In addition, we performed two reference experiments. The positive control was heated in an oven at 100 °C for 1 h, while another set was dispersed and stored at room temperature for 1 h and served as negative control. As there is no alternative way of heating the particle surface locally, the temperature treatment of the hole system at 100 °C ensures that the same temperature is reached at the particle surface, and the polymer is split off. Afterward, the particles were separated from the solvent, and both were analyzed (Figure 5).

To see if the fluorescent polymer could be detached using an AMF-induced retro-Diels–Alder reaction, the fluorescence intensity of the supernatants was measured after inductive heating. The sample heated to 100 °C in an oven exhibited the highest fluorescence intensity, which we assign to a complete detachment of the polymer, as our previous work showed that the majority of the retro-Diels–Alder reaction has taken place after reaching 100 °C.^25^ Consequently, the sample treated at RT showed the lowest fluorescence intensity in the supernatant solution, which correlated to almost no polymer release. Assuming a linear correlation between the intensity of the fluorescence signal and the concentration of the free polymer in solution, one can conclude that 71% of the polymer has detached from the PMO-SPIONs when the AMF has been applied. Our conclusions are supported by IR spectroscopy. The vibrational intensities at 1641 cm^–1^ of the dried particles are compared in Figure 5b; full spectra are given in Supporting Information Figure S6. This vibrational intensity corresponds to the amide I band, the most dominant vibrational band in the FTIR spectrum of the P-HPMA polymer. Consistent with the fluorescence measurement results, the sample stored at room temperature without any further external stimulus exhibited the highest intensity, followed by the AMF sample and 100 °C sample consecutively. According to the IR data, the AMF conditions resulted in a 68% cleavage of the polymer.

To investigate the reversibility of the process, we first detached the polymer using the described AMF method. In a comparative experiment, another sample was stirred for an additional 72 h at 40 °C after this detachment, which are the original conditions for polymer attachment. The particles were then magnetically separated, and the fluorescence intensity of the supernatants was measured. The results (Figure S7) demonstrated that the fluorescence intensity was lower after reattachment of the polymer, suggesting that less detached polymer remained in the solution, indicating successful reattachment to the particles. During exposure, the temperature of the dispersion increased by only Δ*T* = 4.9 °C (Figure 5c), which surprised us. After *t* = 30 min, the temperature does not rise anymore, and *T* = 30 °C should not be sufficient to induce the retro-DA reaction. However, one has to consider that this effect is highly localized and attenuates within the first few nanometers of the surface of the PMO-SPIONs and it is not equal to the temperature in the solution. The combination of the localized heating effect at the particle surface and minimal macroscopic temperature rise allows for potential biomedical applications without the threat of damaging the surrounding tissue.^27,28^

**Figure 6:**
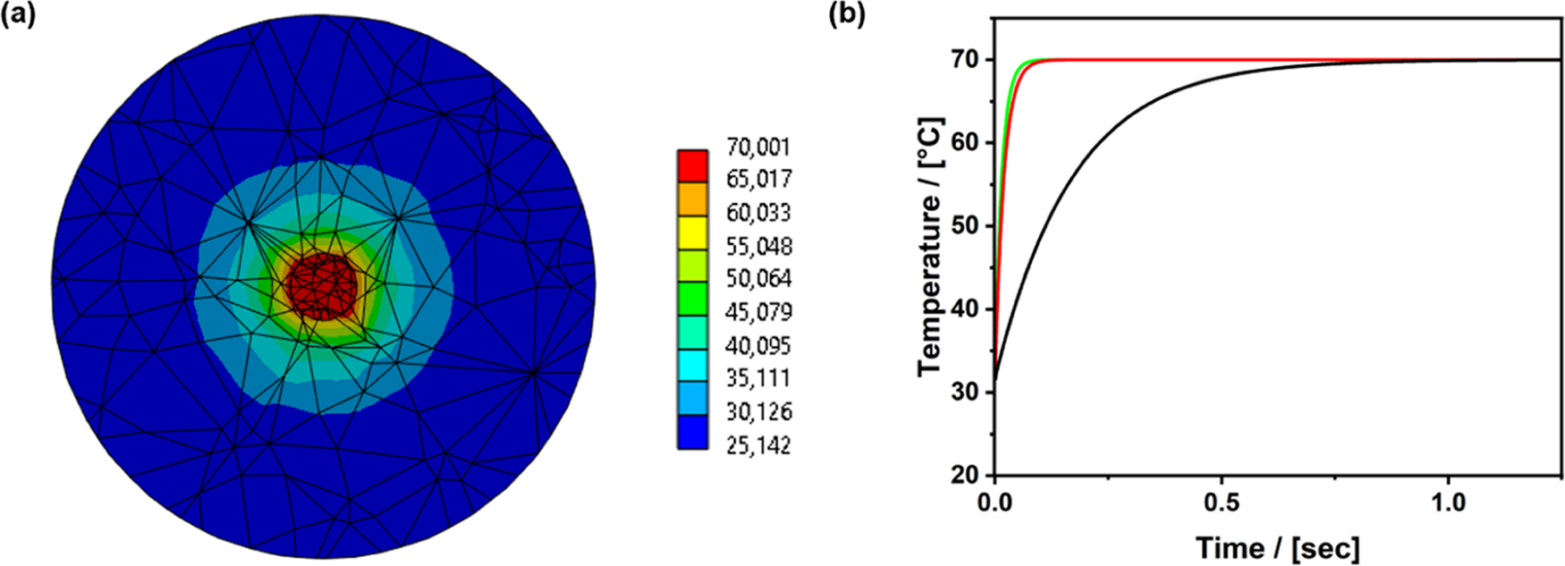
(a) Cut through both spheres and the experimental setup at the start of the simulation, exemplified by PMO B (84 nm). (b) Average temperature throughout the simulation for PMO A (black line), B (red line), and C (green line).

A numerical simulation of the magnetic core–shell nanoparticles was performed by using a transient thermal analysis within the ANSYS program. The geometry was assumed to be perfectly spherical for both the core and the shell. The core is situated perfectly in the center of the shell. For this initial simulation, the core had a diameter set of 11 nm and a shell of 168 (PMO A), 84 (PMO B), and 77 nm (PMO C). The core temperature was set to 70 °C, which is a sensible estimation of the temperatures reached by magnetite cores.^28^ The surrounding temperature was set to 22 °C. For the shell, a thermal conductivity of 0.31 W K^–1^ m^–1^ was selected.^29^ The simulation time was 2 s. Figure 6a shows a cut through both the spheres and the experimental setup at the start of the simulation. The diffusion of temperature is demonstrated. Figure 6b depicts the average temperature throughout the simulation. After approximately 1 s, the whole shell of all of the differently sized particles had a temperature of 70 °C. The PMO C particles, which have the thinnest shell, heat up the fastest, consistent with the results shown in Figure 4b. Thus, the simulation explains the relatively high value for the retro-DA reaction and that the majority of the polymer is released.

## Conclusions

This work presents the direct condensation of a periodic mesoporous organosilica (PMO) shell around superparamagnetic core nanoparticles. By varying the amount of used magnetite cores, the size of the shell can be controlled, and particle sizes between 160 and 70 nm are obtained. Moreover, the post-synthetic surface modification with maleimide groups opens the wide field of cycloaddition reactions. This was used for the thermoreversible attachment of a furan- and fluorescein-modified P-HPMA polymer to the particles via the DA reaction. The particles’ capability of inductive heating was confirmed, and the magnetic settings were optimized, leading to high heating efficiency and beneficial for triggering the retro-Diels–Alder reaction for polymer detachment. Comparative measurements showed that notable amounts of the polymer were detached by AMF. The ability to modulate the particle size combined with efficient polymer attachment and detachment via AMF, where the macroscopic temperature is not strongly increasing, makes them accessible for a broad range of applications, especially in the biomedical field, such as thermally-triggered drug delivery. Furthermore, based on the results of this work, which demonstrate the potential for AMF-induced retro-Diels–Alder between the polymer and core–shell particles, the fabrication of a macroscopic composite is conceivable, wherein the particles cross-link the polymer chains via Diel-Alder. By the AMF-induced disruption of cross-links, the composites’ mechanical properties should change.

## Supporting Information Available

Additional analytical data (PDF)

## Dedication

We dedicate this work to the memory of Peter Behrens, whose invaluable contributions and dedication were instrumental to this research. His legacy and passion for knowledge will continue to inspire us. We thank Florian Klodwig for his technical support in designing the illustrations. We also thank the Warnecke Group from the Department of Otorhinolaryngology-Head and Neck Surgery, Hannover Medical School, 30625 Hanover, Germany, for fluorescence microscopy measurements.
