# Supplementary material for: Magnetic Triggering of Functional Organosilica Filler Particles for Controlling the Thermoreversible Attachment to Polymer Matrices
Source: Langmuir. 2024 Oct 30;40(45):23706–13. doi: 10.1021/acs.langmuir.4c02589 (PMC11562805; doi:10.1021/acs.langmuir.4c02589)
Supplement: Supplementary file 1 — la4c02589_si_001.pdf [file la4c02589_si_001.pdf]

Supporting Information

Magnetic Triggering of Functional Organosilica Filler Particles for Controlling the Thermoreversible Attachment to Polymer Matrices

*Valentin Hagemann[(a,b)], Laura Finck[(c)], Felix Klempt[(d)], Patrick Evers[(e)], Florian Nürnberger[(e)], Irene Morales[(a)], Nina Ehlert[(a,b)], Philipp Junker[(d)], Peter Behrens[(a,b)], Henning Menzel[(c)], Sebastian Polarz[(a),]**

(a) Institute of Inorganic Chemistry, Leibniz-University Hannover, Callinstrasse 9, 30167 Hannover, Germany.

(b) Lower Saxony Center for Biomedical Engineering, Implant Research and Development (NIFE), Stadtfelddamm 34, 30625 Hannover, Germany

(c) Institute for Technical Chemistry, Technical University Braunschweig, Hagenring 30, 38106 Braunschweig, Germany.

(d) Institute of Continuum Mechanics, Leibniz-University Hannover, An der Universität 1, 30823 Garbsen, Germany

(e) Institute of Materials Science, Leibniz-University Hannover, An der Universität 2, 30823 Garbsen, Germany

*Sebastian Polarz: sebastian.polarz@aca.uni-hannover.de

Table of Content

Characterization of magnetite nanoparticles	S-3
TEM images of PMO nanoparticles	S-4
DLS measurements of PMO nanoparticles	S-5
Nitrogen-physisorption of PMO nanoparticles	S-6
Characterization of modified PMO nanoparticles	S-7
FT-IR measurements of PMO nanoparticles after different treatments	S-8
De- and reattachment of polymer to PMO particles	S-9

Number of pages: 9

Number of figures: 7

Number of schemes: 0

Number of tables: 0

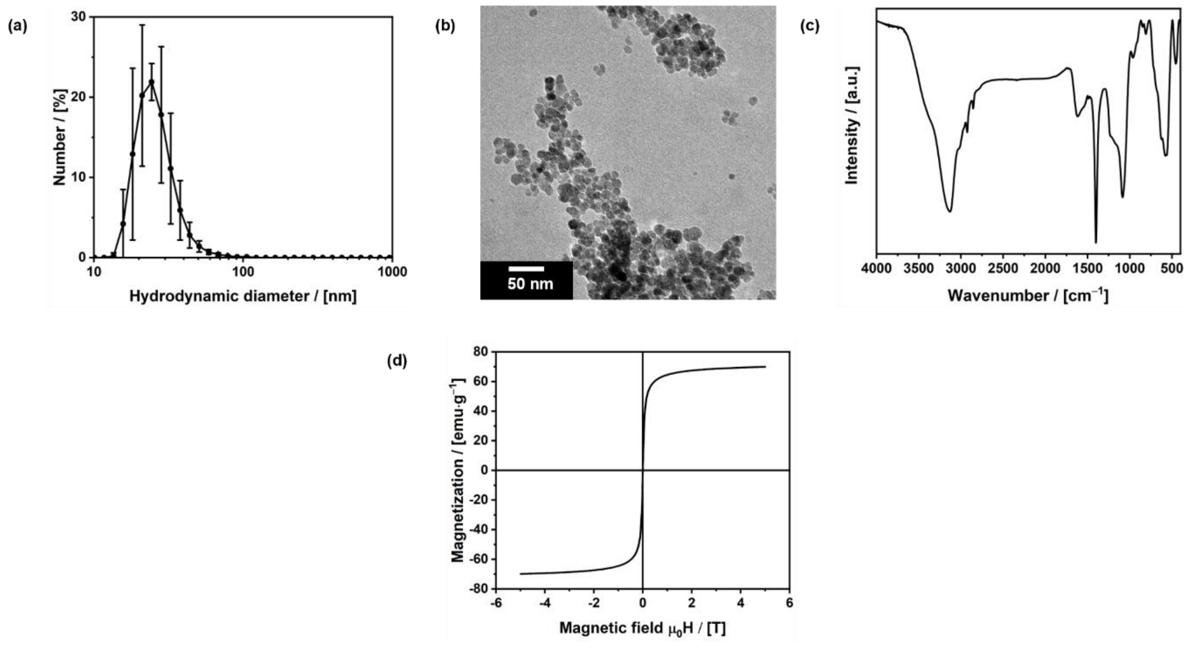

Figure S1. (a) DLS measurement of magnetite cores. (b) TEM image of magnetite cores (11 nm ± 2 nm in diameter). (c) FT-IR spectra of magnetite cores. (d) SQUID measurement of magnetite cores.

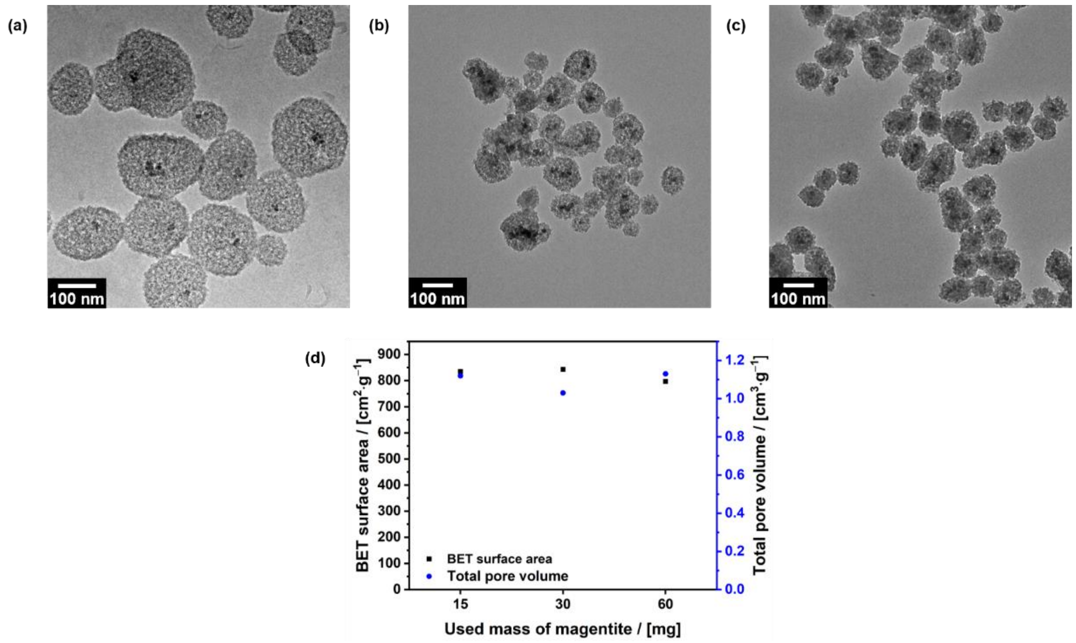

Figure S2. TEM images core-shell nanoparticles PMO A (a), PMO B (b) and PMO C (c). (d) BET surface (black) and total pore volume (blue) of PMO A, B and C.

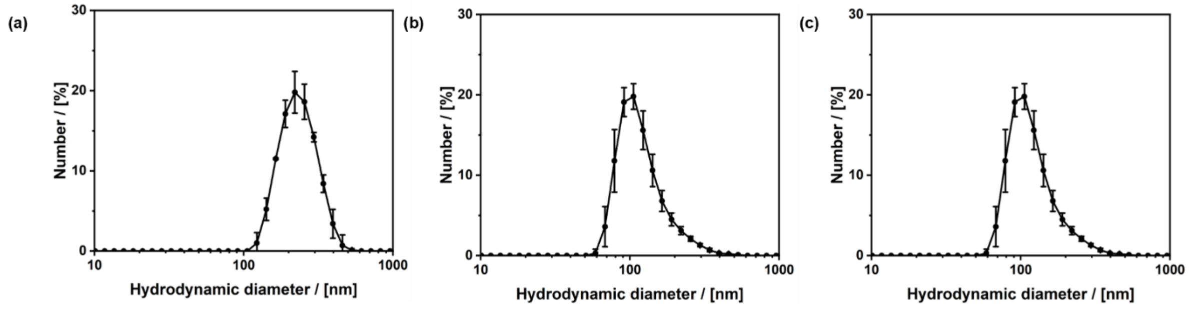

Figure S3. DLS measurements of PMO A (a), PMO B (b) and PMO C (c).

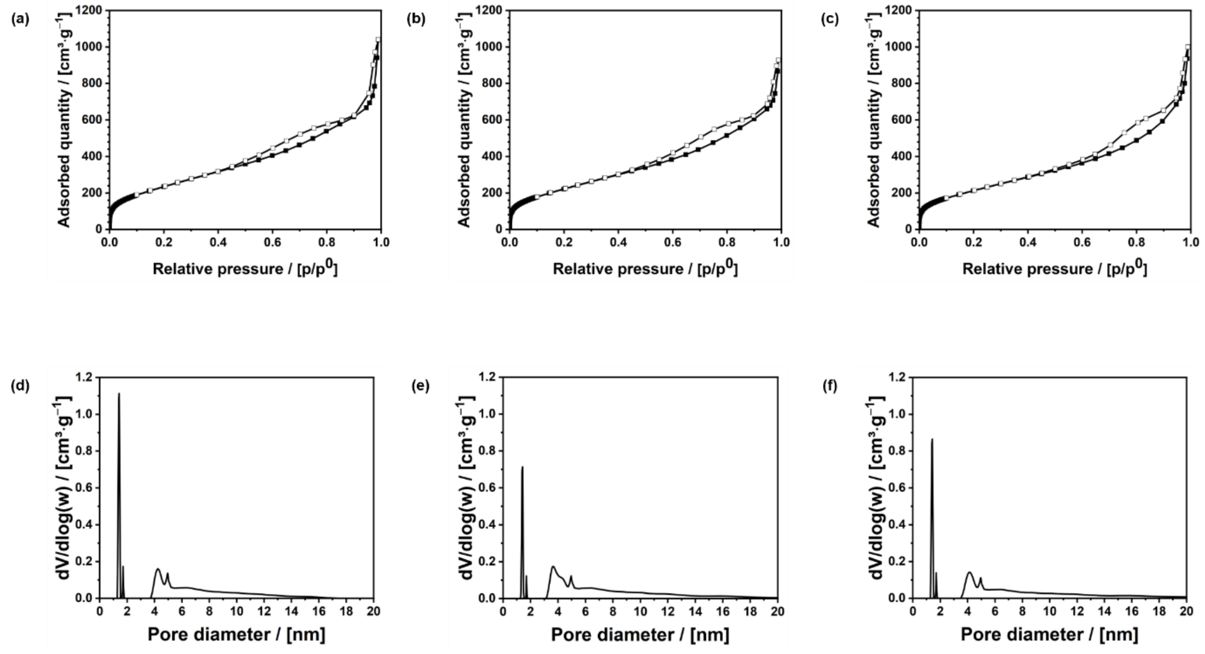

Figure S4. Physisorption measurements. Top: Isotherms of PMO A (a), PMO B (b), PMO C (c). Bottom: Pore size distribution of PMO A (d), PMO B (e), PMO C (f).

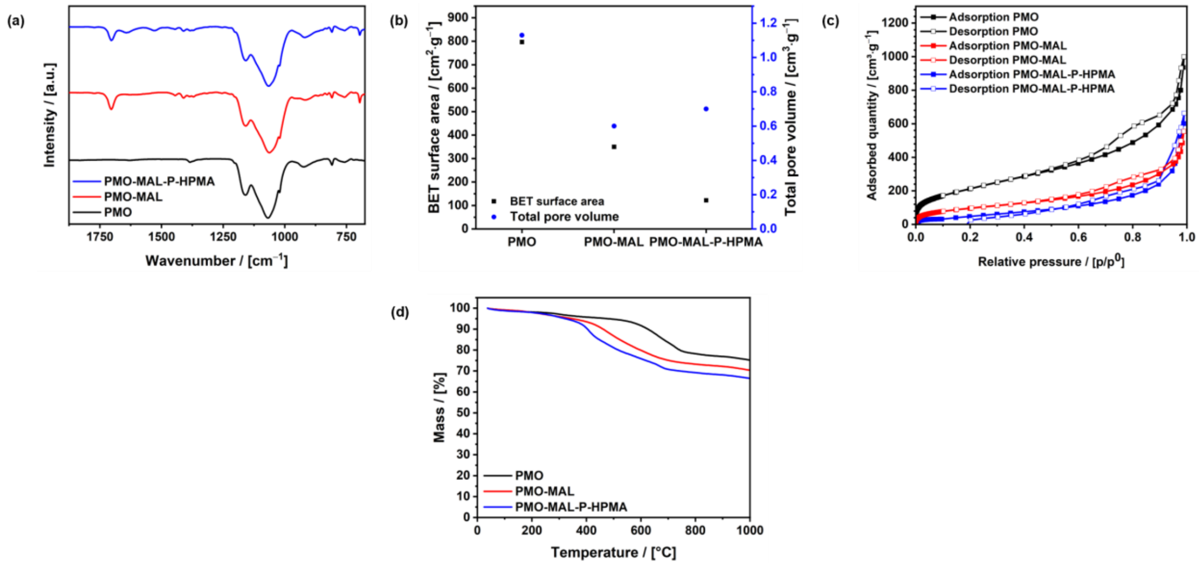

Figure S5. (a) FT-IR spectra of PMOs and after each modification step. (b) Decrease of BET surface and total pore volume after each modification step. (c) Corresponding Isotherms of PMOs and after each modification step. (d) Thermogravimetric measurement of PMOs and after each modification step.

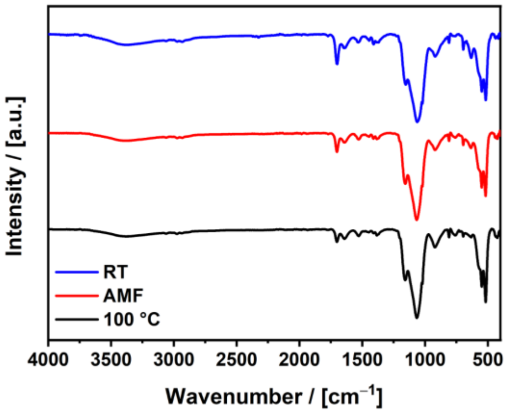

Figure S6. FT-IR spectra for the investigation of the remaining polymer attached to the filler particles after different treatment.

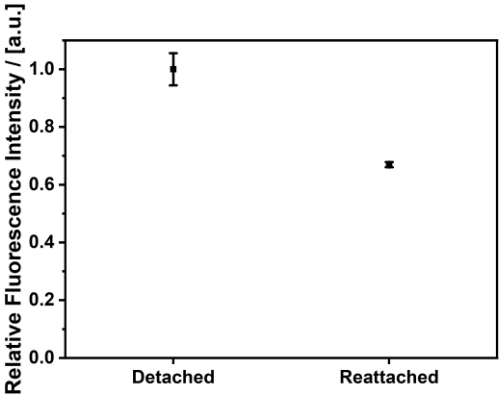

Figure S7. Comparison of relative fluorescence intensity after detachment (set to one) of the polymer by AMF exposure (detached) and after subsequent reattachment of polymer (reattached).
